# Dengue Virus 3 Genotype I in *Aedes aegypti* Mosquitoes and Eggs, Brazil, 2005–2006

**DOI:** 10.3201/eid1606.091000

**Published:** 2010-06

**Authors:** Ana P.P. Vilela, Leandra B. Figueiredo, João R. dos Santos, Álvaro E. Eiras, Cláudio A. Bonjardim, Paulo C.P. Ferreira, Erna G. Kroon

**Affiliations:** Universidade Federal de Minas Gerais, Belo Horizonte, Brazil

**Keywords:** Aedes aegypti, mosquitoes, phylogeny, dengue virus 3, genotype I, ovitrap, mosquitrap, viruses, Brazil, dispatch

## Abstract

Dengue virus type 3 genotype I was detected in Brazil during epidemics in 2002–2004. To confirm this finding, we identified this virus genotype in naturally infected field-caught *Aedes aegypti* mosquitoes and eggs. Results showed usefulness of virus investigations in vectors as a component of active epidemiologic surveillance.

Dengue virus (DENV) is a member of the family *Flaviviridae* and a positive-sense RNA virus. Epidemics caused by the 4 DENV serotypes have emerged as major public health problems in tropical and subtropical regions over the past 20 years (*1*). DENV is transmitted to humans by the bite of an infected mosquito. Female *Aedes aegypti* mosquitoes are the main vector involved in the urban transmission cycle of the virus. *Ae*. *aegypti* is a tropical mosquito that lays its eggs on the walls of containers commonly found in and around homes (*1*). Female mosquitoes remain infectious for their entire lives and have the potential to transmit virus during each human feeding.

Mosquitoes and larvae may be infected by vertical transmission and maintain the virus in nature (*2*). Spread of the mosquito vector and virus has led to a resurgence of dengue fever epidemics and the emergence of dengue hemorrhagic fever (DHF) (*3*). No dengue vaccine is currently available, and dengue control relies solely on vector control. For successful epidemiologic investigations, identification and typing of DENV from field-caught mosquitoes and eggs are needed.

The current epidemiology of dengue in Minas Gerais state, Brazil, is characterized by cocirculation of DENV-1, DENV-2 and DENV-3 serotypes (state and metropolitan health departments, unpub. data). DENV-3 serotype was detected in 2002 and during 2005–2006; this was the most common serotype detected in Minas Gerais in those periods (*4*,*5*). Previous work in our laboratory identified DENV-3 genotype I, which was associated with dengue fever and DHF in Minas Gerais (*6*).

In this study, we confirm circulation of DENV-3 genotype I in naturally infected field-caught *Ae*. *aegypti* mosquitoes and eggs. We show the useful role of virus investigations in mosquitoes and eggs for monitoring DENV circulation.

## The Study

**Figure 1:**
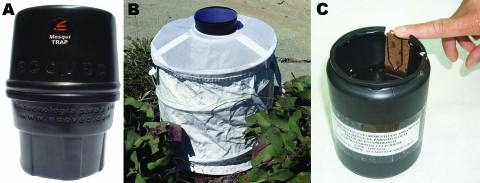
MosquiTRAP version 2.0 (Ecovec Ltd., Belo Horizonte, Brazil) (A), BG-Sentinel trap (Biogents, Regensburg, Germany) (B), and an ovitrap (C) used for obtaining mosquitoes in the northwestern borough of Belo Horizonte, Minas Gerais, Brazil.

Traps designed to catch mosquitoes and eggs were installed in an urban residential area in the northwestern borough of Belo Horizonte, Minas Gerais, Brazil. Since 1998, Belo Horizonte has had a high concentration of dengue cases and high rates of vector infestation. Mosquitoes were obtained during 8 weeks (during October 2005–May 2006). Adult mosquitoes were collected by using 2 capture traps (Figure 1, panels A and B): MosquiTRAP version 2.0 (M trap; Ecovec Ltd., Belo Horizonte, Brazil) (*7*) and the BG-Sentinel trap (BG trap; Biogents, Regensburg, Germany) (*8*). Eggs were collected with an ovitrap (Figure 1, panel C) and hatched into larvae (*9*).

An area of 20 blocks was selected for the survey. In each block, 3 representative houses were selected, and each house received 1 type of trap. All traps were installed outdoors in a shaded area that was also protected from rain. Within each block, traps were rotated so that each house had only 1 trap type for a maximum of 1 week. Mosquitoes collected were identified by sex, trap, and epidemiologic week. Eggs collected were counted, identified by epidemiologic week, and hatched into larvae. All samples were stored at –70°C until analyzed (Table 1).

**Table 1:** *Aedes aegypti* mosquitoes and larvae obtained by using 2 traps and analyzed by PCR, Belo Horizonte, Minas Gerais, Brazil, October 2005–May 2006*

Date	BG-Sentinel trap†						MosquiTRAP version 2.0‡							
	Mosquitoes			Pools			Mosquitoes			Pools			Ovitrap	
	F	M		F	M		F	M		F	M		Larvae	Pools
2005														
Oct	–	–		–	–		3	2		1	1		483	7
Nov	1	–		1	–		–	–		–	–		198	4
Dec	23	21		2	2		30	1		2§	1		760	9
2006														
Jan	–	–		–	–		–	–		–	–		665	13§
Feb	12	13		1	1		8	–		1	–		1,076	22
Mar	16	36		2§	2§		3	–		1§	–		754	14
Apr	14	11		1	1		12	1		1	1		1,004	20
May	11	15		1	1		4	–		1	–		1,298	25
Total	77	96		8	7		60	4		7	3		5,573	101

*–, not available. †Biogents, Regensburg, Germany. ‡Ecovec Ltd., Belo Horizonte, Brazil. §One sample was positive for dengue virus type 3.

Pools of <30 mosquitoes and <50 larvae were triturated on ice, macerated in 300 µL of Leibowitz L15 medium (GIBCO-BRL, Gaithersburg, MD, USA), and centrifuged at 2,000 × *g* for 5 min. RNA from each pool was extracted according to a modified protocol (*10*) and used as template in a reverse transcription–PCR as described (*11*). To amplify the virus genome, a seminested PCR was conducted with a forward primer (5′-CGA GAA ACC GCG TGT CAA C-3′) designed to amplify a 434-nt region that contains the capsid–premembrane (C-prM) gene and a reverse primer as described (*11*). The PCR products were then used for sequencing (MegaBACE sequencer; GE Healthcare, Little Chalfont, UK). Sequences and inferred amino acid sequences were aligned with other available DENV-3 sequences.

Alignments were used to construct midpoint-rooted phylogenetic trees by using the neighbor-joining method. The Tamura-Nei statistical model was implemented by using MEGA4.1 software (Arizona State University, Tempe, AZ, USA) with 1,000 bootstrap replicates. These sequences have been deposited in GenBank (accession nos. GQ330909–11 and GU588695–8).

A total of 237 adult *A. aegypti* mosquitoes (137 females and 100 males) were tested in 25 pools, and 5,573 larvae were tested in 101 pools (Table 1). Fifteen mosquito pools contained only females, and 10 contained only males. Fifteen pools (8 containing females and 7 containing males) were obtained in BG traps, and 10 pools (7 containing females and 3 containing males) were obtained in M traps. Viral RNA was detected in 4 of the 25 *A. aegypti* mosquito pools analyzed. Two positive pools were obtained in M traps, and 2 positive pools were obtained in BG traps. Viral RNA was detected in 1 of 101 larvae pools tested (Table 1). Minimum infection rates were 16.9% for adult mosquitoes and 0.18% for larvae (Table 2). The higher minimum infection rates for mosquitoes could be explained by the lower number of analyzed specimens.

**Table 2:** MIR for dengue virus type 3 in *Aedes aegypti* mosquitoes and larvae, Belo Horizonte, Minas Gerais, Brazil, October 2005–May 2006*

Stage	Positive pools/ analyzed pools	No. specimens analyzed	MIR, %
Male adult	1/10	100	10.0
Female adult	3/15	137	21.9
Both	4/25	237	16.9
Larvae	1/101	5,573	0.18

*MIR, minimum infection rate.

**Figure 2:**
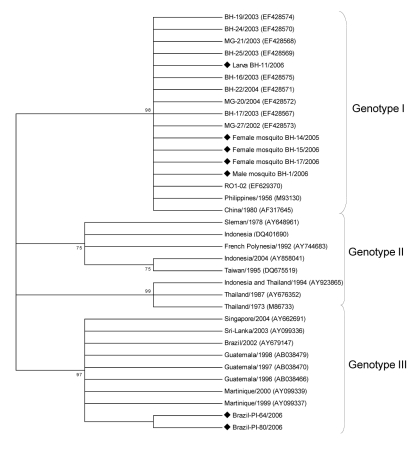
Phylogenetic tree of dengue type 3 serotypes and sequences from *Aedes aegypti* mosquitoes and larvae obtained in Belo Horizonte, Minas Gerais, Brazil. The tree is based on a 434-nt sequence of the capsid–premembrane gene and was generated by using neighbor-joining analysis with the Tamura-Nei model in MEGA4.1 software (Arizona State University, Tempe, AZ, USA). Numbers to the left of the nodes are bootstrap values (1,000 replicates) in support of the grouping to the right. Numbers in parentheses are GenBank accession numbers. Diamonds indicate viruses sequenced in this study.

These data also confirm vertical transmission by detection of DENV-3 in male mosquitoes hatched from eggs. C-prM sequences obtained showed a high degree of similarity to isolates from the Philippines and the People’s Republic of China and recent isolates from Minas Gerais and Rondônia, Brazil (*6*,*12*). Sequence homology values ranged from 98.8% to 99.8%. Phylogenetic analysis confirmed that all 4 samples obtained from adult mosquitoes (female mosquito BH-14/2005, male mosquito BH-1/2006, female mosquito BH-15/2006, and female mosquito BH-17/2006) and larvae (BH-11/2006) were grouped in a well-supported distinct cluster of genotype I (Figure 2).

## Conclusions

Our results suggest that the DENV-3 strains circulating in Minas Gerais, Brazil, in 2005 and 2006 may share a common origin. We identified (*6*) co-circulation of 2 DENV-3 genotypes (I and III) in Brazil (*6*,*12*). DENV-3 isolates detected in Rio de Janeiro, Brazil, during the 2001 and 2002 outbreaks and DENV-3 detected in Latin America were assigned to genotype III, which has been associated with DHF outbreaks (*13*). DENV-3 genotype I identified in outbreaks during 2002–2004 in Minas Gerais (*6*) also showed associations with DHF. This genotype was also identified in Colombia (*14*) and French Guiana (*14*). Sequences showed high similarity to isolates from Southeast Asia and the South Pacific islands and have not been previously reported in South America.

Detection of DENV-3 genotype I in sylvatic animals (*15*) may support the hypothesis of a sylvatic origin for this genotype in South America. Our analysis of C-prM gene sequences from mosquitoes naturally infected with DENV-3 confirmed circulation of genotype I in Minas Gerais. Additionally, our results indicated that dengue virus sequences in mosquitoes and larvae are highly similar to sequences in DENV-3 isolates from patients who are spatially and temporally related. Although vertical transmission has not been routinely determined (*2*), our findings also confirmed that vertical transmission of DENV that may be a major factor in virus prevalence and survival in nature.
